# P2Y_6_ receptors are involved in mediating the effect of inactivated avian influenza virus H5N1 on IL-6 & CXCL8 mRNA expression in respiratory epithelium

**DOI:** 10.1371/journal.pone.0176974

**Published:** 2017-05-11

**Authors:** Nawiya Huipao, Suparerk Borwornpinyo, Suwimon Wiboon-ut, Craig R. Campbell, Il-Ha Lee, Siriphun Hiranyachattada, Chonlaphat Sukasem, Arunee Thitithanyanont, Chumpol Pholpramool, David I. Cook, Anuwat Dinudom

**Affiliations:** 1 Department of Physiology, Faculty of Science, Prince of Songkla University, Songkhla, Thailand; 2 Department of Biotechnology, Faculty of Science, Mahidol University, Bangkok, Thailand; 3 Department of Microbiology, Faculty of Science, Mahidol University, Bangkok, Thailand; 4 Discipline of Physiology, The Bosch Institute, School of Medical Sciences, The University of Sydney, Sydney, Australia; 5 Division of Pharmacogenomics and Personalized Medicine, Department of Pathology, Faculty of Medicine, Ramathibodi Hospital, Mahidol University, Bangkok, Thailand; 6 Department of Physiology, Faculty of Science, Mahidol University, Bangkok, Thailand; University of Geneva, SWITZERLAND

## Abstract

One of the key pathophysiologies of H5N1 infection is excessive proinflammatory cytokine response (cytokine storm) characterized by increases in IFN-β, TNF-α, IL-6, CXCL10, CCL4, CCL2 and CCL5 in the respiratory tract. H5N1-induced cytokine release can occur via an infection-independent mechanism, however, detail of the cellular signaling involved is poorly understood. To elucidate this mechanism, the effect of inactivated (β-propiolactone-treated) H5N1 on the cytokine and chemokine mRNA expression in 16HBE14o- human respiratory epithelial cells was investigated. We found that the inactivated-H5N1 increased mRNA for IL-6 and CXCL8 but not TNF-α, CCL5 or CXCL10. This effect of the inactivated-H5N1 was inhibited by sialic acid receptor inhibitor (α-2,3 sialidase), adenosine diphosphatase (apyrase), P2Y receptor (P2YR) inhibitor (suramin), P2Y_6_R antagonist (MRS2578), phospholipase C inhibitor (U73122), protein kinase C inhibitors (BIM and Gö6976) and cell-permeant Ca^2+^ chelator (BAPTA-AM). Inhibitors of MAPK signaling, including of ERK1/2 (PD98059), p38 MAPK (SB203580) and JNK (SP600125) significantly suppressed the inactivated-H5N1-induced mRNA expression of CXCL8. On the other hand, the inactivated-H5N1-induced mRNA expression of IL-6 was inhibited by SB203580, but not PD98059 or SP600125, whereas SN-50, an inhibitor of NF-κB, inhibited the effect of virus on mRNA expression of both of IL-6 and CXCL8. Taken together, our data suggest that, without infection, inactivated-H5N1 induces mRNA expression of IL-6 and CXCL8 by a mechanism, or mechanisms, requiring interaction between viral hemagglutinin and α-2,3 sialic acid receptors at the cell membrane of host cells, and involves activation of P2Y_6_ purinergic receptors.

## Introduction

H5N1 avian influenza virus infection is a highly fatal disease in humans, with a mortality rate of over 60% [1]. Since the first outbreak in 1997 [2], confirmed cases of H5N1 infection have been reported in several countries [3–6], raising serious concern that the virus may become endemic if it develops ready transmissibility between humans [7]. The fatality risk of patients with H5N1 infection is much higher than those with the human-adapted influenza H1N1 [8]. Initially, H5N1 infected patients develop severe pneumonia, which subsequently progresses to acute respiratory distress syndrome (ARDS) [9,10]. These patients also develop high serum levels of inflammatory cytokines and chemokines [11,12]. Hypercytokinemia (cytokine storm) together with a high level of viral replication is responsible for pathogenesis and a high mortality rate in H5N1 influenza [12].

Human airway epithelium provides the first line of defence against harmful airborne viruses by both detecting presence of pathogens and creating a microenvironment for immune competent cells [13]. In response to harmful pathogens such as influenza A virus, airway epithelial cells deploy innate bioactive molecules, including cytokines, chemokines and interferons, to defend against microbial infection [13]. Cytokines produced by the respiratory epithelial cells, the primary site of infection, instigate local and systemic inflammatory responses, leading to pathophysiological and clinical manifestations of influenza [13,14].

Establishment of influenza A virus infection depends largely on the ability of viruses to attach to respiratory epithelial cells. This is achieved by binding of viral hemagglutinin to sialosaccharide receptors at the host cell membrane [15]. It is well established that hemagglutinin of H1N1 human-adapted influenza virus has preference for sialic acid receptors with NeuAcα2-6Gal linkage (α2-6Sia) expressed conspicuously in human upper respiratory tract, whereas hemagglutinin of H5N1 avian influenza virus has preference for sialic acid receptors with NeuAcα2-3Gal linkage (α2-3Sia) expressed mainly in the lower respiratory tract [16]. During H5N1 influenza virus infection, secretions in the respiratory tract contain cytokines and chemokines at levels higher than those found in the plasma or serum [17–19]. Cytokines initially produced by the respiratory epithelial and immune cells in the airways increase vascular permeability, allowing passage of immune cells from the blood across the endothelial barrier to the infected area, and promote the production of more cytokines and an even greater influx of immune cells [20]. This hypercytokinemia is correlated directly with the severity of the illness and is accountable for the pathogenesis of H5N1 influenza [14,18].

A recent study reported that H5N1 that has been treated with β-propiolactone (BPL), a pharmacological agent that reacts with nucleic acids and proteins and is commonly used for inactivation of DNA and RNA viruses [21], strongly induces expression of cytokines in human respiratory epithelial cells [22]. Moreover, UV-inactivated H5N1 also increased cytokine production in dendritic cells [23]. These studies suggest that H5N1 may be able to initiate an innate immune response in human respiratory cells prior to the onset of infection. The cellular mechanism responsible for this effect of inactivated-H5N1 is currently unknown. In the present study, we test the hypothesis that BPL-inactivated H5N1 can induce the cytokine response in the respiratory epithelium and explore the cellular signaling mechanism underlying this infection-independent effect of H5N1.

## Materials and methods

### Virus preparation

Influenza viruses, H5N1 (A/open-billed stork/Nakhonsawan/BBD0104F/04) and H1N1 (A/California/07/09(H1N1)-X179A) were produced in embryonated chicken eggs (ECEs) obtained from the Bureau of Veterinary Biologics, Department of Livestock Development, Nakhonratchasima, Thailand. The virus was grown in ECEs (5 plaque-forming unit (PFU)/egg) incubated at 37°C for 37–38 hr. The allantoic and amniotic fluids were harvested from ECEs, pooled, and centrifuged at 4,000 rpm for 5 min at 4°C to remove debris. The supernatants were collected to measure hemagglutinin (HA) and PFU titers and subsequently filtered through an 0.8 μm-filter membrane. The virus was concentrated by filtering with a Labscale Tangential Flow Filtration System (Millipore) with a 1,000 KDa MWCO membrane cassette (PelliconXL, Millipore) and inactivated with 0.1% β-propiolactone (BPL) at 37°C for 2 hr. Inactivated virus was purified by filtering with a 1,000 KDa MWCO membrane cassette (Pellicon, Millipore) and subsequently filtered through a 0.45 μm membrane filter. Complete inactivation was verified by absent cytopathic effects in cell culture and lack of plaques in the supernatant after adding purified inactivated H5N1 to MDCK cell culture for 2 passages. The quantity of hemagglutinin was determined by hemagglutinin ELISA kit (Sino Biological) using a protocol provided by the manufacturer. The final concentration was adjusted to 20 μg/ml hemagglutinin by diluting with culture media. Allantoic fluid used for control experiments throughout this study was prepared from non-inoculated ECEs with the same protocol as described above for preparation of inactivated viruses.

### Cell cultures

The human bronchial epithelial cell line (16HBE14o-), originally developed from human bronchial-surface epithelial cells [24], was a gift from Prof. Dr. Dieter C. Gruenert (University of California San Francisco, San Francisco, California) and the Madin-Darby canine kidney (MDCK) cell line was from American Type Culture Collection (ATCC). 16HBE14o- cells retain properties of differentiated airway epithelial cells [24]. Both cell types were maintained at 37°C in a humidified atmosphere with 5% CO_2_ in Minimum Essential Medium (MEM, Gibco) supplemented with 10% (vol/vol) fetal bovine serum, 2 mM L-glutamine and 100 U/ml penicillin-streptomycin. Supplements were purchased from J R Scientific. For mRNA analysis, cells were seeded onto a 24-well culture plate (Corning) at a density of 2×10^5^ cells/well. Twenty-four hours after seeding, cells were washed twice with PBS and thereafter incubated in serum-free medium. Cells were allowed to reach 80–90% confluence before being used for experiments.

After incubation overnight in serum-free medium, cells were exposed to inactivated virus at a concentration equivalent to 20 μg/ml hemagglutinin. The incubated cells were harvested 3, 6 and 12 hrs after exposure, as appropriate, for analysis of cytokine gene expression. Pharmacological inhibitors and enzymes were added to the culture medium 1 hr prior to exposure to the inactivated virus. These include, at final concentration, 2 μM cytochalasin D (Sigma), 2 U/ml apyrase (Sigma), 100 μM suramin (Sigma), 20 μM MRS2179 (Tocris), 10 μM MRS2578 (Sigma), 10 μM U73122 (Tocris), 50 μM BAPTA-AM (Molecular Probes), 1μM BIM (Tocris), 10 μM Gö6976 (Tocris), 10 μM SB203580 (Tocris), 50 μM PD98059 (Tocris), 10 μM SP600125 (Tocris), 10 μM SN-50 (Calbiochem) and 1 U/ml α2–3 specific sialidase (TaKaRa).

### RNA Extraction and quantitative real-time PCR (qRT-PCR)

Total RNA was extracted from cells using TRIzol Reagent (Invitrogen) according to the manufacturer’s instructions. After treatment with DNase I (Thermo Scientific), 1 μg of RNA was reverse transcribed to cDNA using an iScript^TM^ select cDNA synthesis kit (Bio-Rad) in a thermal cycler (model PTC-100TM, MJ Research, Inc.). The following qRT-PCR primers were used to allow calculation of relative mRNA of cytokines of interest: TNF-α (NM_000594.3) forward, AGCCCATGTTGTAGCAAACC; reverse, TGAGGTACAGGCCCTCTGAT; IL-6 (NM_000600.3) forward, GAACTCCTTCTCCACAAGCG; reverse, GCGGCTACATCTTTGGAATC; CXCL8 (NM_000584.3) forward, CCAACACAGAAATTATTGTAAAGC; reverse, TGAATTCTCAGCCCTCTTCAA; CCL5 (NM_002985.2) forward, TACCATGAAGGTCTCCGC; reverse, GACAAAGACGACTGCTGG; CXCL10 (NM_001565.3) forward, TTCAAGGAGTACCTCTCTCTAG; reverse, CTGGATTCAGACATCTCTTCTC; P2Y_6_R (NM_001277208.1) forward, CCACAGGCATCCAGCGTAAC; reverse AGGAAGCCGATGACAGTGAGAG and GAPDH (NM_001289746.1) forward, ATGACATCAAGAAGGTGGTG; reverse, CATACCAGGAAATGAGCTTG. qRT-PCR reactions were performed in duplicate using SYBR select master mix (Applied Biosystems) on a CFX96 Touch™ Real-Time PCR Detection System (Bio-Rad). PCR conditions were 50°C for 2 min followed by 95°C for 2 min before 40 cycles of 95°C for 15 sec and 60°C for 1 min. GAPDH was used as an endogenous control for normalization. The comparative Δ-Δ C_T_ method was used to determine the expression level of the respective genes.

### Transfection with small interfering RNA (siRNA)

16HBE14o- cells were trypsinized before being transfected with 100 pmol P2Y_6_R siRNA (Qiagen) using Lipofectamine^TM^ 2000 (Invitrogen) according to the manufacturer’s instructions. Transfected cells were seeded on a 24-well culture plate at a density of 2.5×10^5^ cells/well and grown in MEM culture medium without antibiotics. AllStars siRNA (Qiagen) was used as a negative control and was introduced into the cells using the same protocol. One day after transfection and seeding, cells were washed with PBS and grown in serum-free medium for 18–24 hrs before being treated with inactivated-H5N1.

### IL-6 and CXCL8 protein secretion assay

16HBE14o- cells were seeded on a 12-well culture plate at a density of 4×10^5^ cells/well. Two days later, culture medium was replaced with fresh medium containing inactivated-H5N1 (20 μg/ml H5 hemagglutinin). A further thirty-six hours later, the medium was harvested and analyzed for IL-6 and CXCL8 by a magnetic bead-based multiplex quantitative cytokine immunoassay (BioPlex) using a protocol provided by the BioRad. Briefly, antibody–conjugated beads were added to individual wells of a 96-well filter plate and adhered using vacuum filtration. After washing with a washing buffer, 50 μl of prediluted standard or culture medium was added to each well and the plate shaken at 300 rpm for 30 min at room temperature. Thereafter, prediluted multiplex biotin conjugated detection antibody was added, followed by a 30 min incubation on the shaker. After washing, prediluted streptavidin-conjugated PE was added to each well and incubated for 10 min. Wells were washed with washing buffer before the BioPlex assay buffer was added. After 5 min incubation, concentrations of each cytokine were determined using a BioPlex 200 instrument equipped with BioPlex Manager V 5.0 software (BioRad). Each sample was run in duplicate and the mean value reported. Data were normalized against control untreated groups and obtained from at least 3 independent experiments.

### Protein extraction and immunoblot analysis

16HBE14o- cells were seeded on a 12-well culture plate at a density of 4×10^5^ cells/well. One day after seeding, cells were washed twice with PBS and incubated in serum-free medium for 18–24 hrs before being used in experiments. After incubation in serum-free medium, cells were exposed to inactivated-H5N1 at a concentration equivalent to 20 μg/ml H5 hemagglutinin. The incubated cells were harvested at 30 min after being treated with the virus, as appropriate for the immunoblot analysis. Total protein was extracted in RIPA buffer (Merck) containing protease inhibitor (Roche) and phosphatase inhibitor (Calbiochem). Protein concentrations were determined by Bradford protein assay (BioRad) at the wavelength 595 nm, using bovine serum albumin as the standard. Equal amounts of protein samples (30 μg) were mixed with 2× sample-loading buffer, denatured by boiling for 5 min and centrifuged at 14,000 rpm for 10 min at 4°C. Samples were separated by SDS-PAGE (12% polyacrylamide). Following electrophoretic separation, proteins were transferred to polyvinylidene difluoride membranes and incubated for 1 hr at room temperature in 5% fat-free milk dissolved in Tris-buffered saline with Tween 20 (TBST). After blocking, membranes were incubated overnight at 4°C with primary antibody against phospho-ERK1/2 at 1:2000 dilution, ERK1/2 at 1:2000 dilution, phospho-p38 MAPK at 1:1000 dilution or p38 MAPK at 1:2000 dilution. All antibodies were purchased from Cell Signaling Technology. After four washes with TBST (5 min each), membranes were incubated with horseradish peroxidase-conjugated goat anti-rabbit IgG (GE Health Science) at 1:5000 dilution. Membranes were washed several times with TBST and developed using Enhanced Chemiluminescence (ECL) reagent (Merck Millipore), then exposed to green X-ray film (Kodak).

### Immunofluorescence techniques

For sialic acid staining, 16HBE14o- or MDCK cells were seeded onto coverslips in 24-well culture plates at a density of 1×10^5^ cells/well and 1x10^4^ cells/well, respectively. Two days after seeding, the culture medium was removed and cells were washed twice with cold PBS and fixed with 4% paraformaldehyde for 20 min. Subsequently, cells were washed 3 times with PBS and incubated in PBS containing 10 μg/ml MAL I (*Maackia amurensis* lectin), for α-2,3 sialic acid receptor, or SNA (*Sambucus nigra* lectin), for α-2,6 sialic acid receptor, for 1 hr. Cells were then rinsed 3 times with PBS before the coverslips were mounted in antifade reagent containing DAPI (Thermo Fisher Scientific) to stain the nuclei. A negative control was obtained by incubating cells with PBS in the absence of primary antibody. Fluorescent imaging was performed by confocal laser-scanning microscope (FV1000; Olympus). Cells were excited at 358 and 495 nm and emission detected at 461 and 515 nm to evaluate DAPI and FITC fluorescence, respectively. To remove sialic acid receptors from the cell surface, cells were treated with 1 U/ml α-2,3 sialidase for 1 hr at 37°C prior to sialic acid staining.

### Statistical analysis

All data are expressed as mean ± standard error (SEM) from at least 3 sets of experiments. Statistical difference was assessed using one-way analysis of variance (ANOVA) with Student-Newman-Keuls post-hoc test to compare fold change difference and unpaired Student’s *t*-test to compare % change of mRNA expression level. *p* < 0.05 was considered to be statistically significant.

## Results

**Fig 1:**
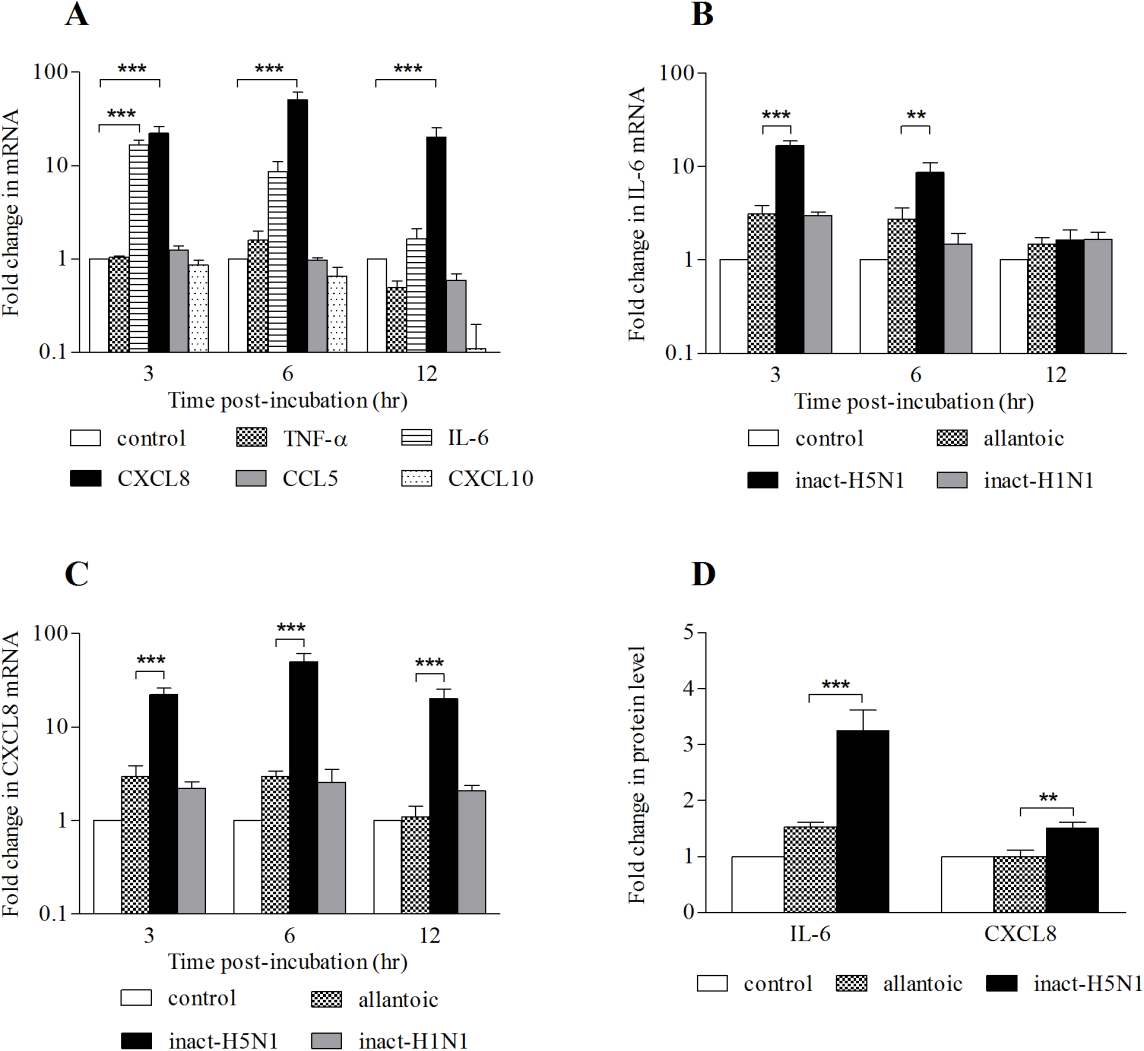
Inactivated-H5N1 increases mRNA of IL-6 & CXCL8. ***(****A)* Fold changes (log scale) in mRNA level of TNF-α, IL-6, CXCL8, CCL5 or CXCL10 in 16HBE14o- cells treated with inactivated-H5N1 (20 μg/ml hemagglutinin) or untreated cells (control) for 3 hr, 6 hr or 12 hr. *(B)* Fold changes in mRNA expression level of IL-6 and *(C)* CXCL8 in untreated cells (control), allantoic fluid, inactivated-H5N1 or inactivated-H1N1. *(D)* Fold changes in protein concentrations of IL-6 and CXCL8 in cell-free culture medium in untreated cells (control) or treated for 36 hr with allantoic fluid or inactivated-H5N1 were analyzed by Bio-plex assay. Data were normalized against control untreated groups. Values are means ± SEM from at least 3 sets of experiments. *, ** and *** indicates *p* < 0.05, *p <* 0.01 and *p* < 0.001, respectively (one-way ANOVA with Student-Newman-Keuls post-hoc test), compared to untreated control *(A)* or allantoic fluid *(B*, *C*, and *D)*.

To show the cytokine gene expression profile of respiratory epithelial cells in response to inactivated-H5N1, 16HBE14o- cells were incubated with inactivated virus for 3, 6 or 12 hrs prior to qRT-PCR analysis of expression of the cytokine and chemokine genes of interest. We could not observe any significant change in mRNA expression of TNF-α, CCL5 or CXCL10 within 12 hrs of exposure of the cells to inactivated-H5N1 (Fig 1A). Of the 5 cytokine and chemokine mRNAs investigated, only IL-6 and CXCL8 were significantly elevated. Expression of IL-6 mRNA was more than 15-fold of that of the allantoic fluid-treated cells at 3 hr after exposure to the inactivated-H5N1 and declined to a level not significantly different from control within 12 hrs (Fig 1B). On the other hand, the expression of CXCL8 mRNA increased to 50-fold greater than that of allantoic fluid-treated cells at 6 hr after treatment and remained significantly higher than that of the allantoic fluid-treated cells 12 hours after the exposure (Fig 1C). An effect of inactivated-H5N1 on the level of IL-6 and CXCL8 proteins in cell culture medium was then determined 36 hrs after exposure. Results showed that the level of IL-6 in culture medium bathing cells treated with inactivated-H5N1 was 2-fold greater than that of medium bathing cells treated with allantoic fluid. Furthermore, inactivated-H5N1 treatment increased CXCL8 in the culture medium significantly (Fig 1D).

**Fig 2:**
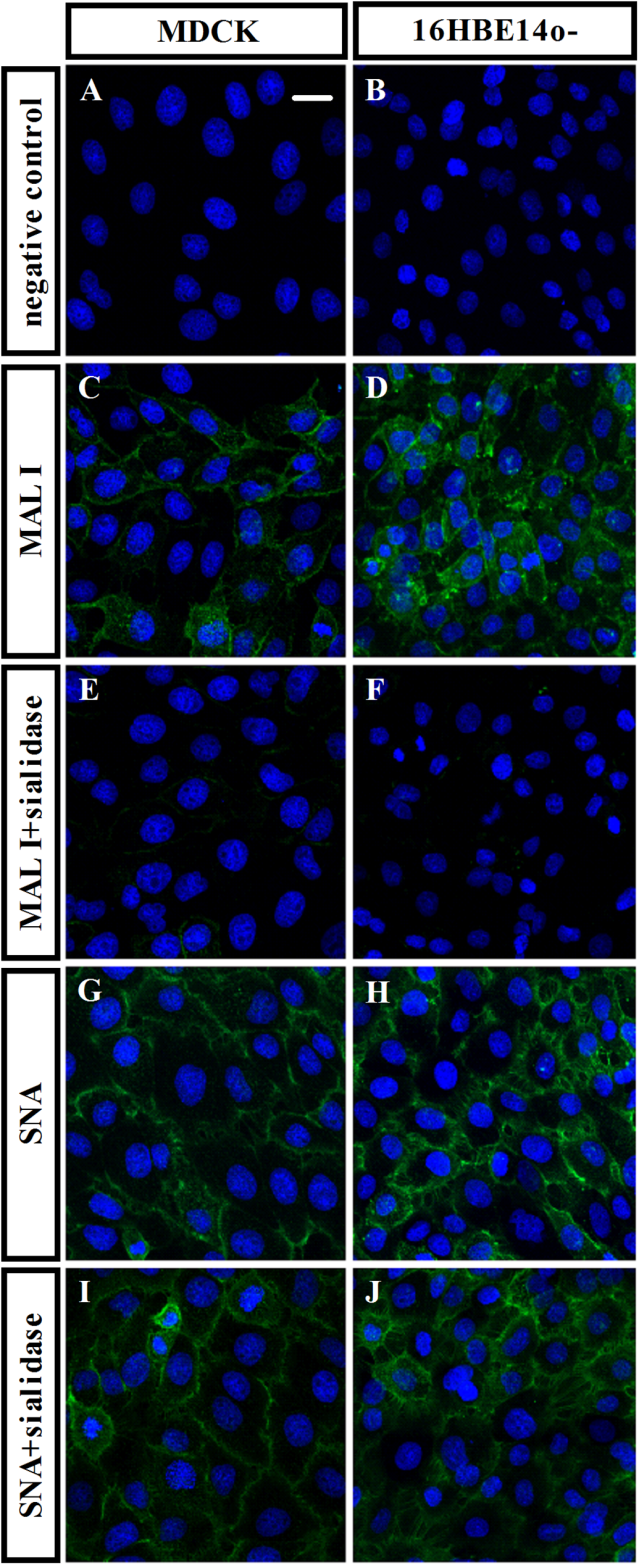
α-2,3 sialic acid receptor is expressed in 16HBE14o- cells. Representative confocal images (800×) of 16HBE14o- cells *(B*, *D*, *F*, *H and J)* or MDCK cells *(A*, *C*, *E*, *G and I)* treated with DAPI (blue), to stain for nuclei, together with fluorescein-coupled antibody directed against α-2,3 sialic acid receptors, (green; *C*, *D*, *E and F*) or fluorescein-coupled antibody directed against α-2,6 sialic acid receptors, (green; *G*, *H*, *I and J*) or without fluorescein-lectin staining (negative control; *A and B*). The effect of sialidase on sialic acid receptors expression was performed by incubating cells with 1 U/ml sialidase 1 hr before stained with MAL I (MAL I+sialidase; *E and F*) or SNA (SNA+sialidase; *I and J*). Scale bar is 20 μm.

**Fig 3:**
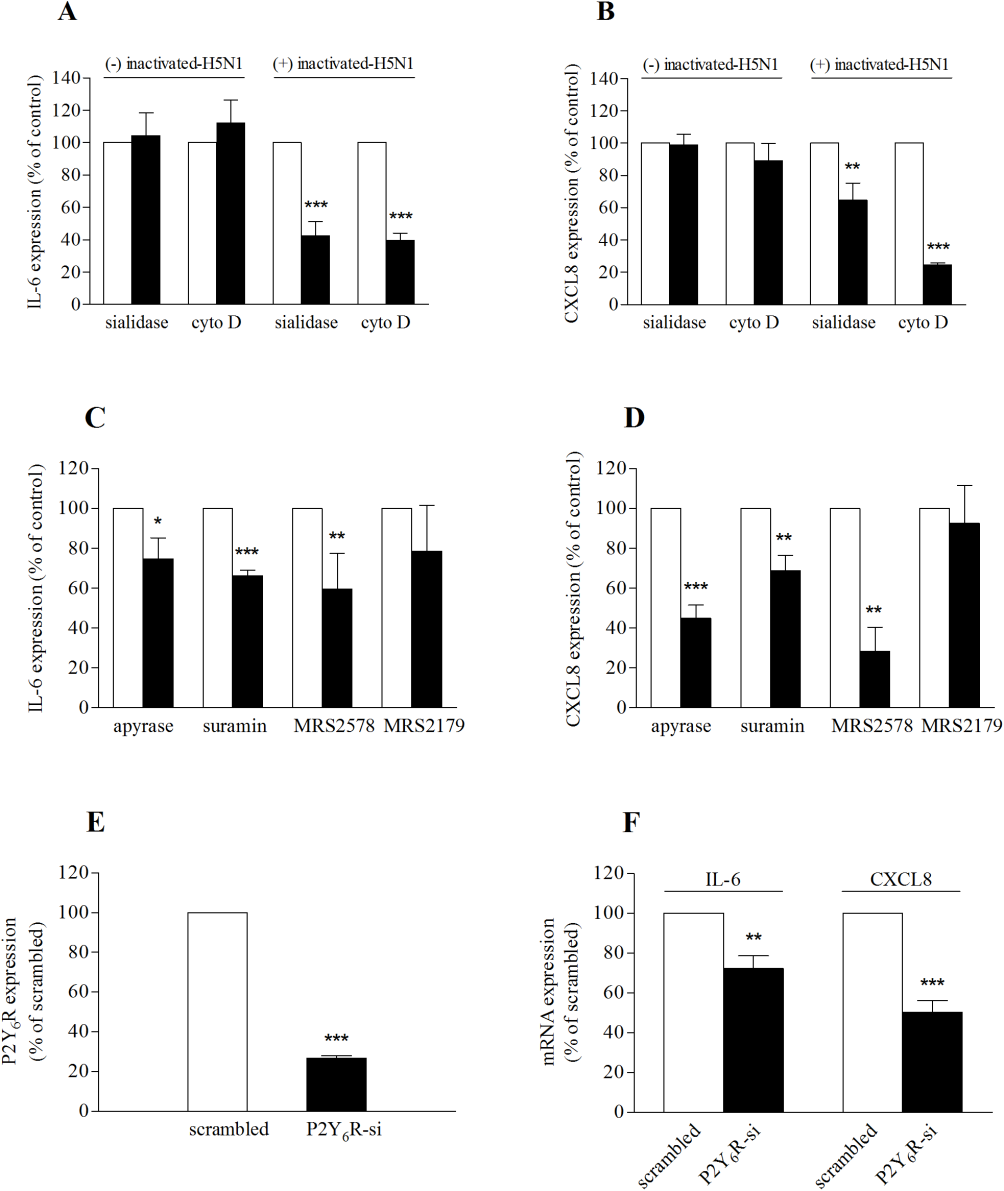
Effect of inactivated-H5N1 is mediated via P2Y_6_ purinoceptors. The effect of inactivated-H5N1 on IL-6 *(A and C)* and CXCL8 *(B and D)* mRNA expression. 16HBE14o- cells were incubated with vehicle (dimethyl sulfoxide (DMSO) or H_2_O as appropriate) as control, 1 U/ml sialidase, 2 μM cytochalasin D (cytoD) *(A and B)* or with apyrase (2 U/ml), suramin (100 μM), MRS2578 (10 μM) or MRS2179 (20 μM) *(C and D)*, for 1 hr before exposure to inactivated-H5N1. Three hours after treatment, cells were harvested for qRT-PCR. Data were normalized with corresponding control groups from the same batch of cells treated with inactivated-H5N1 (open bars) and reported as a percentage. *(A and B)* cells were treated (+) or untreated (-) with inactivated-H5N1 as indicated. *(E)* 16HBE14o- cells were transfected with siRNA directed against P2Y_6_R or a scrambled-siRNA (100 pmol). Forty-eight hours after transfection, cells were exposed to inactivated-H5N1 for 3 hr before being harvested and the mRNA expression of P2Y_6_R analyzed. *(F)* Cells in *(E)* were incubated with inactivated-H5N1 for 3 hr before being harvested and analyzed for mRNA expression of IL-6 and CXCL8. Data were normalized with a group of cells treated with allantoic fluid, as described, and reported as a percentage of normalized scrambled-siRNA treated group. Values are means ± SEM from at least 3 sets of experiments. *, ** and *** indicate *p* < 0.05, *p* < 0.01 and *p* < 0.001, respectively (unpaired Student’s *t*-test).

Hemagglutinin (HA), an important protein of influenza A viruses, is essential for interaction between influenza viruses and targeted host cells [25]. H5 hemagglutinin has been shown to bind α-2,3 sialic acid receptors at the host cell membrane [7]. To investigate the role of H5HA in inactivated-H5N1-mediated cytokine responses, we first confirmed the presence of α-2,3 sialic acid receptors in 16HBE14o- cells. Cells were labelled with MAL I, a leucoagglutinin that binds to glycoproteins with α-2,3 sialic acid linkages, or with SNA, a leucoagglutinin that binds to glycoprotein with α-2,6 sialic acid linkages (Fig 2); both were tagged with fluorescein (FITC). MDCK cells, previously reported to express both the α-2,3 and α-2,6 sialic acid receptors [26], were used as a positive control (Fig 2A, 2C, 2E, 2G and 2I). Both MAL I (Fig 2D) and SNA (Fig 2H) were detected, suggesting the presence of both α-2,3 and α-2,6 sialic acid receptors in 16HBE14o- cells. To investigate whether the effect of inactivated-H5N1 on IL-6 and CXCL8 mRNA requires functional α-2,3 sialic acid receptors at the host cell membrane, we treated 16HBE14o- cells for 1 hr with 1 U/ml α-2,3 sialidase, an enzyme that hydrolyses α-2,3 glycosidic linkages of terminal sialic residues of glycoproteins, before exposing cells to inactivated-H5N1. The specificity of the α-2,3 sialidase on the α-2,3 sialic acid receptor was confirmed by the observation that the α-2,3 sialic acid receptor in 16HBE14o- cells was diminished following treatment with the α-2,3 sialidase (Fig 2F). Sialidase treatment, however, had no effect on the expression of α-2,6 sialic acid receptors (Fig 2J). Expression of IL-6 and CXCL8 was analyzed three hours after cells were exposed to the sialidase. We found that mRNA expression of IL-6 (Fig 3A) and that of CXCL8 (Fig 3B) in these cells were significantly reduced relative to cells not treated with sialidase. It should be noted that sialidase has no effect on the mRNA expression of IL-6 and CXCL8 in cells that have not been treated with the inactivated virus (Fig 3A and 3B). These data suggest that activity of the α-2,3 sialic acid receptors is required for inactivated-H5N1 to induce IL-6 and CXCL8 responses in respiratory epithelium.

There are several lines of evidence suggesting that the cytoskeleton of the host cells plays an important role in influenza A virus infection, including mediating virus entry into the epithelial cells [27], and is involved in cellular mechanisms that regulate host’s antiviral and cell death signals [28]. To determine whether the actin cytoskeleton is involved in the mechanism by which the inactivated-H5N1 mediates the cytokine response, we treated 16HBE14o- cells with 2 μM cytochalasin D, a potent inhibitor of actin polymerization, for 1 hr before exposing the cells to inactivated-H5N1. We found that cytochalasin D treatment significantly attenuated the effect of inactivated-H5N1 on IL-6 and CXCL8 mRNA (Fig 3A and 3B).

Purinergic signaling is known to be involved in host-defence mechanisms of the respiratory epithelium. Previous studies suggested that cytokine production in respiratory cells in response to infection may be activated by purinergic stimulation [29,30]. To investigate the involvement of purinergic signaling systems in the mechanisms by which inactivated-H5N1 stimulates cytokine gene expression, 16HBE14o- cells were treated with apyrase (2U/ml), an enzyme that catalyzes hydrolysis of nucleotide triphosphates and nucleotide diphosphates. Exposure to apyrase attenuated the effect of inactivated-H5N1 on mRNA expression of IL-6 (Fig 3C) and CXCL8 mRNA (Fig 3D). Blocking purinergic receptors with suramin (100 μM) also reduced the effect of inactivated-H5N1 on IL-6 and CXCL8 mRNA expression (Fig 3C and 3D). Furthermore, blocking P2Y_6_ receptors (P2Y_6_R) with MRS2578 attenuated the effect of inactivated-H5N1 on mRNA expression of IL-6 and CXCL8. On the other hand, MRS2179, a blocker of P2Y_1_ receptors (P2Y_1_R), was without effect. Together, these data suggest that inactivated-H5N1 may increase IL-6 and CXCL8 mRNA by a mechanism that involves activation of P2Y_6_Rs. To confirm this hypothesis, expression of P2Y_6_Rs in 16HBE14o- was knocked-down by specific siRNA, and the efficiency of the siRNA in suppressing mRNA expression of P2Y_6_R was confirmed by qRT-PCR (Fig 3E). Consistent with our inhibitor study that suggests the role of P2Y_6_R signaling on H5N1-mediated IL-6 and CXCL8 mRNA expression, the effect of inactivated-H5N1 on gene expression of IL-6 and CXCL8 (Fig 3F) was significantly reduced in cells in which P2Y_6_R expression had been inhibited.

**Fig 4:**
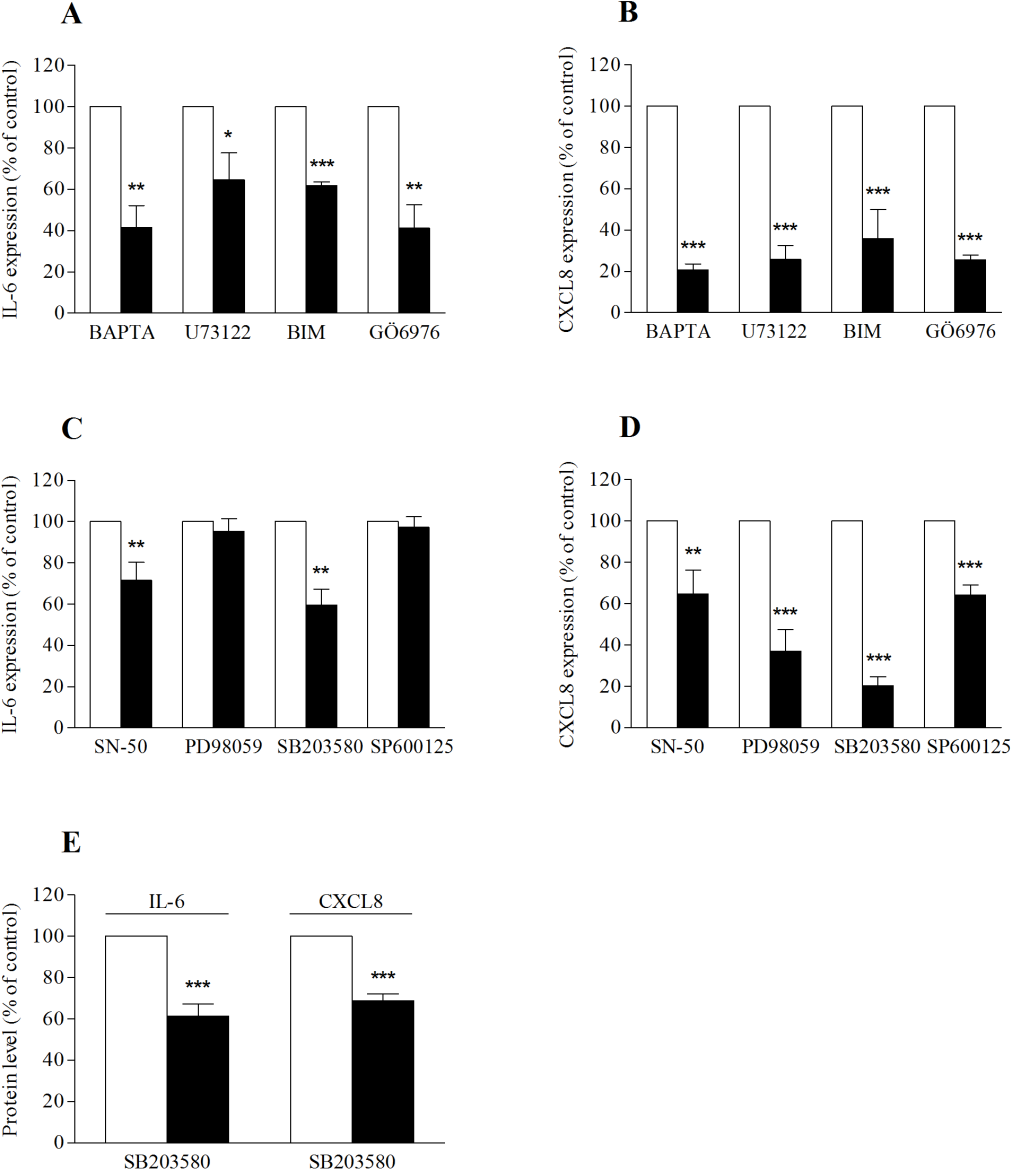
The cellular signaling mechanism by which inactivated-H5N1 increases mRNA for IL-6 and CXCL8 involves intracellular Ca^2+^, PLC, PKC, ERK1/2 and p38 MAPK. 16HBE14o- cells were incubated with BAPTA-AM (50 μM), U73122 (10 μM), BIM (1 μM), Gö6976 (10 μM) ***(****A and B)*, or with SN-50 (10 μM), PD98059 (50 μM), SB203580 (10 μM) or SP600125 (10 μM) *(C and D)*, for 1 hr before being exposed to inactivated-H5N1. Three hours later, mRNA expression of IL-6 *(A and C)* or that of CXCL8 *(B and D)* was analyzed. All data were normalized with corresponding control groups treated with inactivated-H5N1 (open bars) and reported as a percentage. *(E)* Cells were treated with SB203580 (10 μM) for 1 hr before incubation with inactivated-H5N1 (20 μg/ml hemagglutinin). Thirty-six hours later, the cell-free culture medium was harvested and analyzed for the presence of IL-6 and CXCL8 proteins as described in Fig 1. Data are presented as a percentage of control (treated with inactivated-H5N1, open bar). Values are means μ SEM from at least 3 sets of experiments. *, ** and *** indicates *p* < 0.05, *p* < 0.01 and *p* < 0.001 compared with control, respectively (unpaired Student’s *t*-test).

P2Y_6_ receptors are known to mediate their effect via phospholipase C (PLC) and intracellular Ca^2+^ signaling [31]. To further determine the cellular signaling mechanism by which inactivated-H5N1 regulates cytokine mRNA expression, 16HBE14o- cells were pre-treated with a membrane-permeable Ca^2+^ chelator, BAPTA-AM. In the presence of BAPTA-AM, the stimulatory effect of inactivated-H5N1 on IL-6 (Fig 4A) and CXCL8 (Fig 4B) gene expression was reduced by 60% and 80%, respectively. Moreover, the effect of inactivated-H5N1 was attenuated in cells pre-treated with a PLC inhibitor (U73122) and protein kinase C (PKC) inhibitors (BIM and Gö6976) (Fig 4A and 4B). It should be noted that none of BAPTA-AM, U73122, BIM or Gö6976 had any effect on the mRNA expression of IL-6 or CXCL8 in cells untreated with inactivated-H5N1 (S1 Fig).

**Fig 5:**
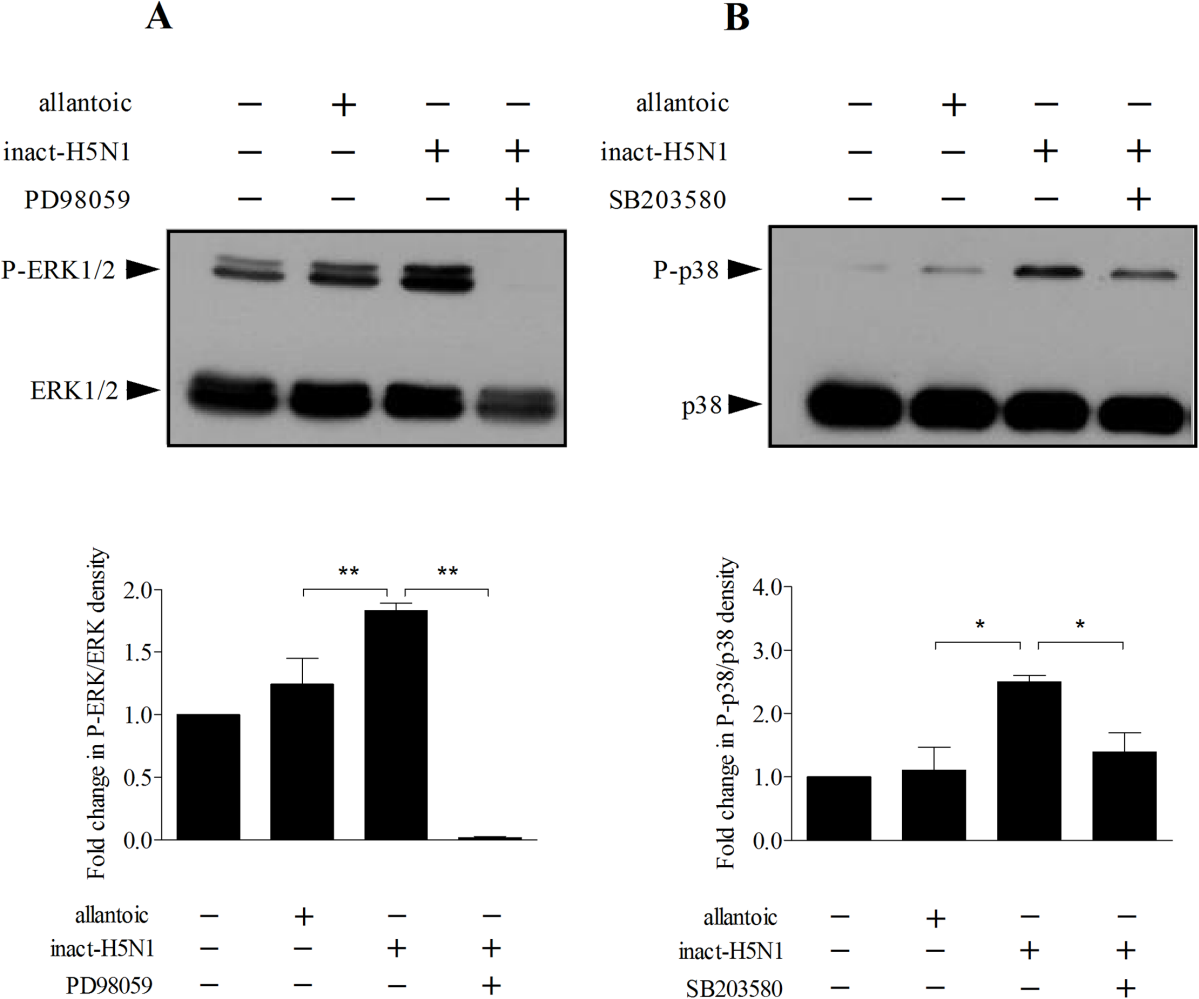
Inactivated-H5N1 increases phosphorylation of ERK1/2 and p38 MAPK. Immunoblot analysis of ERK1/2 *(A)* and p38 MAPK *(B)* protein expression in 16HBE14o- cells treated with inactivated-H5N1. Cells were untreated or treated with PD98059 (50 μM) or SB203580 (10 μM) as appropriate. One hour later, cells were treated for 30 min with vehicle, allantoic fluid or inactivated-H5N1 before being harvested for immunoblot analysis for the presence of ERK1/2, phospho-ERK1/2 (P-ERK1/2), p38 MAPK or phospho-p38 MAPK (P-p38). Upper panel, representative gels for immunoblot analysis of ERK1/2 and phosphorylated ERK1/2 (P-ERK1/2) *(A)* or p38 MAPK and phosphorylated p38 MAPK (P-p38) *(B)*. Lower panel, quantitative analysis of the amount of P-ERK1/2 (*A*) or P-p38 MAPK *(B)*, expressed as fold change, using ImageJ software (NIH). Values are means ± SEM from at least 3 sets of experiments. * and *** indicates *p* < 0.05 and *p* < 0.001, respectively (one-way ANOVA with Student-Newman-Keuls post-hoc test).

Next, the activity of NF-κB in 16HBE14o- cells was inhibited by pharmacological blocker, SN-50 (10 μM). As shown in Fig 4C and 4D, the cytokine mRNA expression responses to inactivated-H5N1 in cells treated with SN-50 were significantly lower than those of control, untreated cells. To investigate whether MAP kinases are involved in the signaling, activity of ERK1/2, p38 and JNK kinases in 16HBE14o- cells were inhibited by specific pharmacological blockers, PD98059, SB203580 and SP600125, respectively. We found that the effect of inactivated-H5N1 on IL-6 mRNA was attenuated by SB203580 but not by PD98059 or SP600125 (Fig 4C). In contrast, the effect of inactivated-H5N1 on mRNA expression of CXCL8 was strongly attenuated by both PD98059 and SB203580 whereas the inhibitory effect of SP600125 on inactivated-H5N1-induced CXCL8 mRNA expression was smaller compared to those of SB203580 and PD98059 (Fig 4D). To determine whether blocking activity of p38 MAPK would attenuate the effect of inactivated-H5N1 on the production of IL-6 and CXCL8, cells were pre-treated with SB203580 (10 μM) for 1 hr before exposure to inactivated-H5N1. Cells were then incubated with inactivated-H5N1 and SB203580 for 36 hr before levels of IL-6 and CXCL8 in the culture medium were determined. IL-6 and CXCL8 in cells treated with virus and SB203580 were significantly lower than those of cells in culture medium treated with only inactivated-H5N1 (Fig 4E). Finally, to confirm that the effect of inactivated-H5N1 on cytokine production is due to activation of p38 and ERK1/2 in 16HBE14o- cells immunoblot analyses were performed. We found that inactivated-H5N1 increased phosphorylation of ERK1/2 (Fig 5A) and p38 MAPK (Fig 5B) in 16HBE14o- cells, and that the effects of inactivated-H5N1 on phosphorylation of ERK1/2 and p38 MAPK were inhibited by PD98059 and SB203580, respectively (Fig 5A and 5B). It should be noted that none of SN-50, SB203580, PD98059, or SP600125 had any effect on mRNA expression of IL-6 or that of CXCL8 (S1 Fig) in cells untreated with inactivated-H5N1, suggesting that, at the concentration used in our experiments, these pharmacological blockers have no effect on mRNA transcription of these cytokines.

## Discussion

Clinical features that are prominent in H5N1 infected patients, including acute respiratory distress syndrome and multiple organ dysfunction, have previously been associated with exacerbation of the proinflammatory cytokine response, hypercytokinemia, an important hallmark of H5N1 infection [5]. It has been postulated that, as the primary target of influenza viruses, an initial viral replication in the respiratory tract may be responsible for the onset of hypercytokinemia [32]. In response to H5N1 infection, bronchial epithelial cells increase mRNA of cytokines and chemokines, including CCL5, CXCL10, IL-6, CXCL8 and TNF-α [33]. Moreover, H7N9, another strain of avian influenza, also increases cytokine production in human respiratory cells [34]. In the present study, we treated 16HBE14o- cells with an inactivated-H5N1 strain, A/open-billed stork/Nakhonsawan/BBD0104F/04. This strain of virus shares common unique genetic sequences and similar biological characteristics with that of H5N1 virus collected, in Thailand, from infected human and others avian species [35]. In 16HBE14o- cells, mRNA for IL-6 and CXCL8 was upregulated within 3 hr following exposure to inactivated-H5N1. In contrast to the cytokine response to live H5N1 virus [33], we observed no alteration of expression of mRNA for CCL5, CXCL10 or TNF-α by the inactivated virus. The difference in the profile of the cytokine response between cells infected with live H5N1 and those exposed to inactivated virus suggests that components of the viral envelope can initiate expression of IL-6 and CXCL8 independent of infection or viral replication. Consequently, the signaling mechanisms by which inactivated-H5N1 activates production of IL-6 and CXCL8 in respiratory cells are likely to differ from those that activate production of TNF-α, CXCL10 and CCL5.

Inhibition of α-2,3 sialic acid receptors by specific sialidase attenuated the stimulatory effect of inactivated-H5N1 on expression of IL-6 and CXCL8. Thus, it is likely that interaction between inactivated-H5N1 and the sialic acid receptors at the cell membrane is involved in the initiation of the IL-6 and CXCL8 mRNA expression response. It has been reported that interaction between H5N1 and α-2,3 sialic acid receptors may generate a distinct inflammatory response in host cells [23]. A previous study suggested that H5 hemagglutinin may be directly responsible for an increase in the expression of CXCL10 and IRF-1 genes in A549 human pulmonary epithelial cells [36]. Our data, however, do not suggest a direct role of H5 hemagglutinin since two commercial recombinant H5 hemagglutinins (Immune Technology, Cat #IT-003-0051p, and Protein Science Corp, Cat #3006) failed to increase mRNA expression of IL-6 and CXCL8 in 16HBE14o- cells (S2 Fig). Binding of H5 hemagglutinin to the α-2,3 sialic acid receptor in stabilizing the virus at the host cell membrane may permit another component(s) of the virus to activate the appropriate cellular signaling pathways and lead to increased production of IL-6 and CXCL8 observed in our study. Interestingly, cytochalasin D inhibited inactivated-H5N1-induced IL-6 (Fig 3A) and CXCL8 (Fig 3B) mRNA expression. This finding suggests that the actin cytoskeleton is involved in the cellular mechanism that regulates the cytokine response to the inactivated-H5N1 in respiratory epithelial cells.

Influenza A viruses are known to increase nucleotide release the respiratory epithelium [37,38], and the activation of P2Y receptors by these nucleotides plays an important role in the negative effect of influenza A viruses on transepithelial Na^+^ absorption and alveolar fluid clearance [37]. It has been reported that 16HBE14o- cells express several P2Y receptor subtypes, including P2Y_1_, P2Y_2_, P2Y_4_ and P2Y_6_ receptors [38]. In line with the role for purinergic signaling in the inflammatory response to H5N1, we found that the effect of inactivated-H5N1 on mRNA for IL-6 and CXCL8 was attenuated by suramin, a broad-spectrum antagonist of P2 purinergic receptors, and by apyrase, an enzyme that catalyzes hydrolysis of nucleotide triphosphates and diphosphates. Since MRS2578, a specific antagonist of P2Y_6_ receptors (P2Y_6_R), but not MRS2179, a specific antagonist of P2Y_1_Rs, attenuated this effect of inactivated-H5N1, P2Y_6_R signaling is most likely to play an important part in the infection-independent response of respiratory epithelia to H5N1. In agreement with this finding, previous reports have suggested that activation of P2Y_6_ receptors produces the proinflammatory cytokine response in monocytes [39], microglia [40], keratinocytes [41], intestinal [42], lung epithelial cells [39,43,44] and 16HBE14o- cells [45]. In addition, P2Y_6_R signaling has been shown to participate in inflammatory responses that elevate IL-6 [41,46,47] and CXCL8 [41–44,47,48] production. P2Y_6_R is a G_q/11_-coupled receptor that generates its cellular signaling via PLC, PKC, and elevation of intracellular Ca^2+^concentration ([Ca^2+^]_i_) [31,49]. Consistent with this, the effect of the inactivated-H5N1 on cytokine mRNA expression was attenuated by U73122, a phospholipase C inhibitor, BIM and Gö6976, both of which are inhibitors of PKC, or when [Ca^2+^]_i_ was sequestered with BAPTA-AM. Together these findings suggest the involvement of PLC, PKC and [Ca^2+^]_i_ in the H5N1-mediated cellular signaling mechanism that regulates IL-6 and CXCL8 responses.

Several lines of evidence suggested that MAP kinases play a key role in the regulation of cytokine production. ERK1/2 and p38 MAPK are known to regulate production of CXCL8 in cystic fibrosis lung epithelial cells [50] and alveolar macrophages [51]. In addition, the cellular signaling pathway by which *Chlamydia* induces CXCL8 mRNA involves ERK1/2 [52], as does the mechanism by which H9N2 increases production of IL-1β, IL-6 and CXCL8 in chicken macrophages [53].

It has also been reported that p38 MAPK is involved in the signaling pathway by which influenza A virus subtype H3N2 activates mRNA expression of the chemotactic cytokine, CCL5, in human bronchial epithelial cells [54]. Further, H5N1 has also been reported to increase production of TNF-α [55] and CCL2, TFN-β and IFN-λ1 [56] in human macrophages in a p38 MAPK-dependent manner. Our data show that the inactivated-H5N1 increased phosphorylation of both ERK1/2 and p38 MAPK in 16HBE14o- cells (Fig 5). It seems likely that p38 MAPK, but not ERK1/2 or JNK, forms part of the signal transduction mechanism employed by H5N1 to upregulate production of IL-6 in the respiratory epithelium since the IL-6 mRNA expression response to inactivated virus is attenuated by SB203580 but not by PD98059 or SP600125 (Fig 4C and 4D). Given that SB203580 inhibited approximately 40% of inactivated-H5N1-mediated IL-6 mRNA expression (Fig 4C), the possibility that other cellular signaling mechanism(s) unrelated to p38 MAPK may contribute to H5N1-induced IL-6 production cannot be excluded. On the other hand, the signaling mechanism that upregulates production of CXCL8 may involve all three MAP kinases, i.e. p38 MAPK, ERK1/2 and JNK. Both SB203580 and PD968059, however, have stronger inhibitory effects on inactivated-H5N1-mediated CXCL8 mRNA expression than that of SP600125. These data suggest a more prominent role for both p38 MAPK and ERK1/2 in regulation of mRNA for CXCL8 by inactivated-H5N1. Consistent with our findings, previous studies in polarized cells reported that CXCL8, but not IL-6, secretion may be regulated by an ERK1/2-dependent pathway, whereas p38 MAPK signaling regulates secretion of both IL-6 and CXCL8 [57]. Interestingly, SN-50, an NF-κB blocker, inhibits approximately 30% of the inactivated-H5N1-induced IL-6 and CXCL8 mRNA response. The apparent lack of a strong inhibitory effect of SN-50 suggests that other transcription factor(s) may have an important role to play. On this note, a MAPK-dependent but NF-κB-independent pathway has been reported to mediate the proinflammatory cytokine response to viral infections [58].

There is a growing body of evidence to suggest that influenza A virus can regulate functions of respiratory epithelial cells by infection-independent mechanisms. For example, a previous study reported that replication-deficient H1N1 influenza virus inhibited Na^+^ absorption in mouse trachea by a mechanism that involves PKC [59]. In addition, the influenza matrix protein 2 is known to inhibit the activity of epithelial Na^+^ channels [60] and expression of cystic fibrosis transmembrane conductance regulator Cl^-^ channels [61,62] in respiratory epithelial cells. Here we demonstrate that replication-deficient H5N1 influenza virus can induce production of IL-6 and CXCL8 in respiratory epithelial cells, suggesting that this effect of H5N1 is likely to be triggered by viral structural proteins. Activation of IL-6 and CXCL8 production by inactivated-H5N1 requires binding of viral particles to α-2,3 sialic acid receptors at the host cell membrane and stimulation of P2Y_6_ receptor by nucleotides released from respiratory epithelial cells. The cellular mechanisms underlying this effect of virus involve PLC, PKC and MAP kinases. Since cytokine responses by respiratory epithelial cells play an important role in initiating the innate immune response to influenza virus infection, our data suggest that pharmacological inhibitors of PLC, PKC and MAP kinases may be valuable therapeutic tools that can be used to attenuate or at least delay onset of hypercytokinemia commonly found in H5N1-infected patients.

## Supporting information

S1 Fig Effect of pharmacological blockers on mRNA expression of IL-6 and CXCL8. Fold change in mRNA expression level of IL-6 *(A and C)* and CXCL8 *(B and D)* in 16HBE14o- cells treated for 3 hr with vehicle (0.1% DMSO) as control or with BAPTA-AM (50 μM), U73122 (10 μM), BIM (1 μM) or Gö6976 (10 μM) ***(****A and B)*, or with SN-50 (10 μM), PD98059 (50 μM), SB203580 (10 μM) or SP600125 (10 μM) *(C and D)*. Data were normalised against the corresponding control. Values are means ± SEM from at least 3 sets of experiments. No statistical difference was detected compared to the control cells (one-way ANOVA with Student-Newman-Keuls post-hoc test).
(TIF)

S2 Fig Effect of commercial recombinant H5 haemagglutinin on mRNA expression of IL-6 and CXCL8. Fold change in mRNA expression level of IL-6 *(A)* and CXCL8 *(B)* in 16HBE14o- cells treated for 3 hr with commercial recombinant H5 hemagglutinins, H5HA-1 (Immune Technology, Cat #IT-003-0051p) and H5HA-2 (Protein Science Corp, Cat #3006). Data were normalized against control untreated 16HBE14o- cells. Values are means ± SEM. from at least 3 sets of experiments. No statistical difference was detected compared to the control cells (one-way ANOVA with Student-Newman-Keuls post-hoc test).
(TIF)
