# Supplementary figures and images for: P2Y6 receptors are involved in mediating the effect of inactivated avian influenza virus H5N1 on IL-6 & CXCL8 mRNA expression in respiratory epithelium
Source: PLoS One. 2017 May 11;12(5):e0176974. doi: 10.1371/journal.pone.0176974 (PMC5426635; doi:10.1371/journal.pone.0176974)

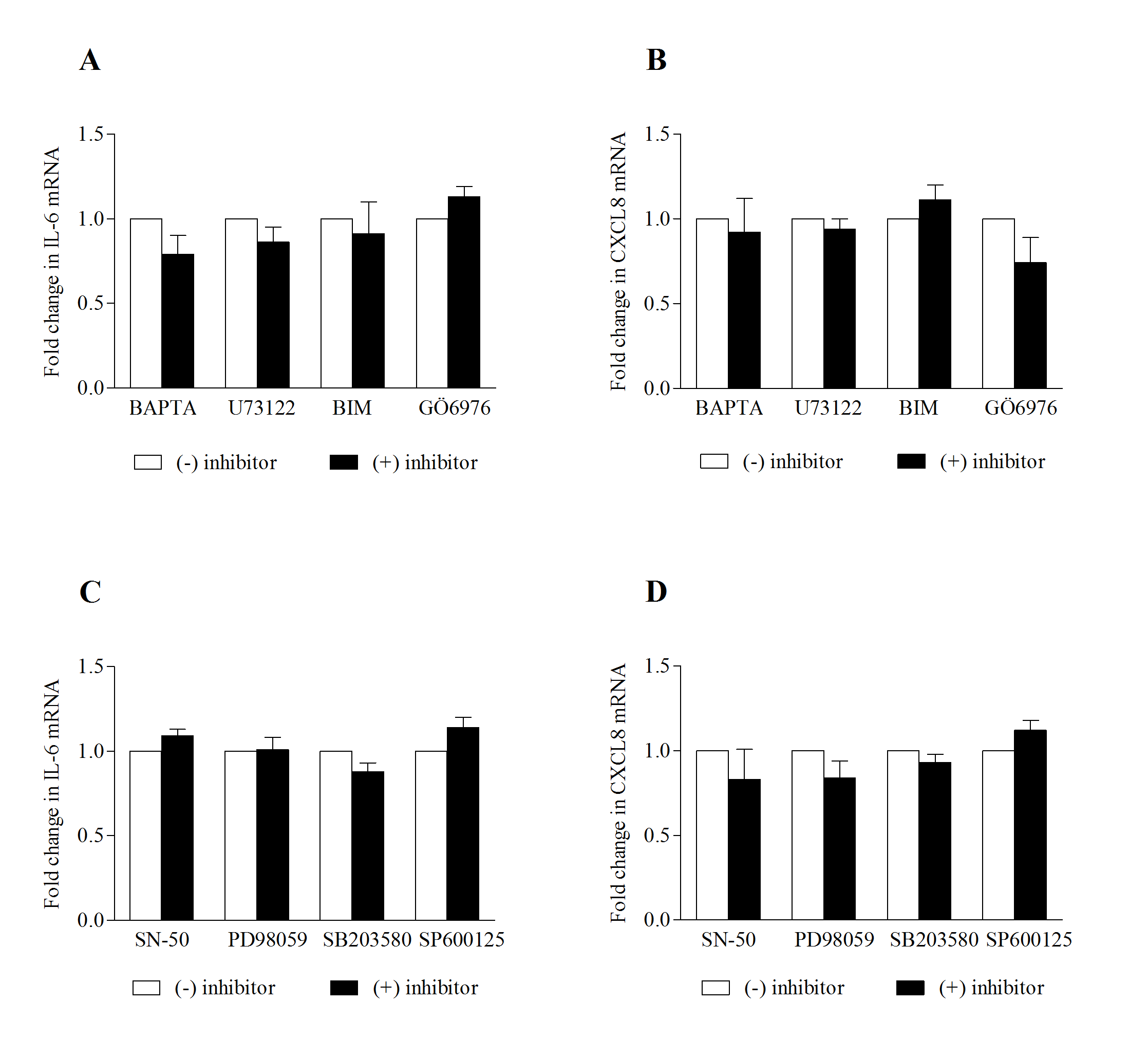

Supplement: S1 Fig — Fold change in mRNA expression level of IL-6 (A and C) and CXCL8 (B and D) in 16HBE14o- cells treated for 3 hr with vehicle (0.1% DMSO) as control or with BAPTA-AM (50 μM), U73122 (10 μM), BIM (1 μM) or Gö6976 (10 μM) (A and B), or with SN-50 (10 μM), PD98059 (50 μM), SB203580 (10 μM) or SP600125 (10 μM) (C and D). Data were normalised against the corresponding control. Values are means ± SEM from at least 3 sets of experiments. No statistical difference was detected compared to the control cells (one-way ANOVA with Student-Newman-Keuls post-hoc test). (TIF) [file pone.0176974.s001.tif]

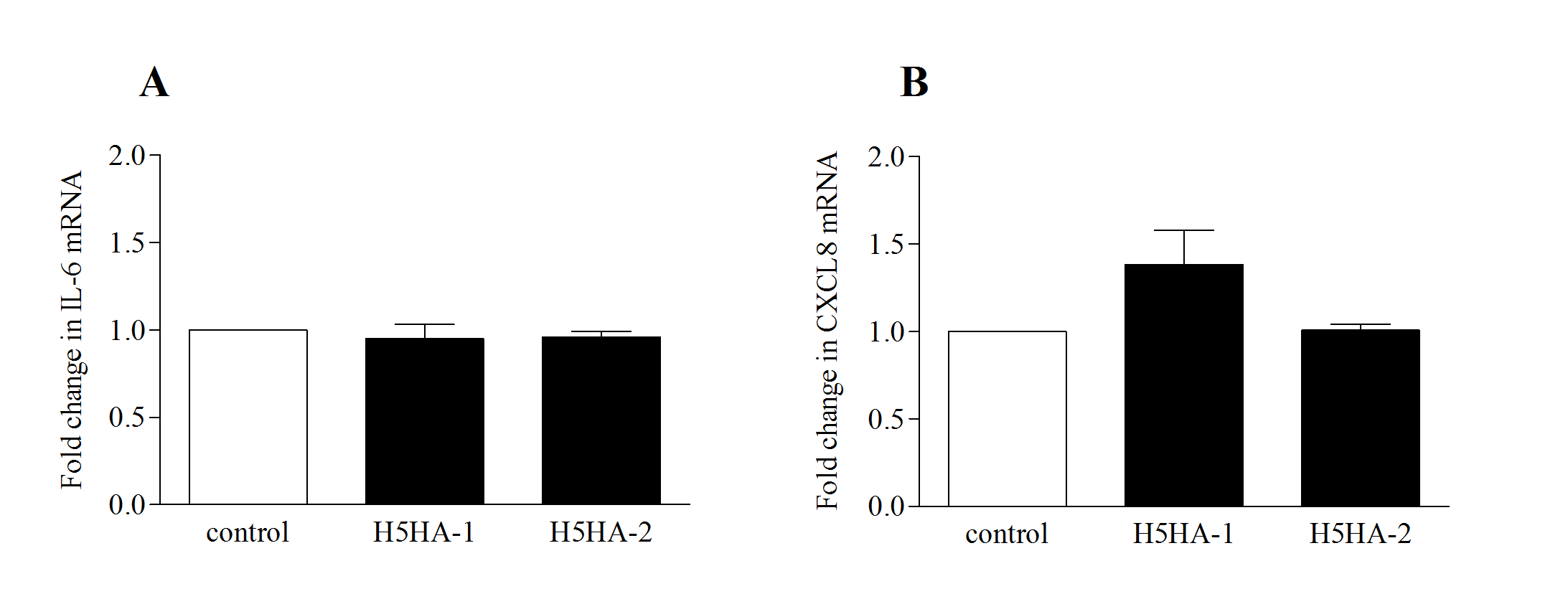

Supplement: S2 Fig — Fold change in mRNA expression level of IL-6 (A) and CXCL8 (B) in 16HBE14o- cells treated for 3 hr with commercial recombinant H5 hemagglutinins, H5HA-1 (Immune Technology, Cat #IT-003-0051p) and H5HA-2 (Protein Science Corp, Cat #3006). Data were normalized against control untreated 16HBE14o- cells. Values are means ± SEM. from at least 3 sets of experiments. No statistical difference was detected compared to the control cells (one-way ANOVA with Student-Newman-Keuls post-hoc test). (TIF) [file pone.0176974.s002.tif]
